# Supplementary material for: Large-scale protein-protein post-translational modification extraction with distant supervision and confidence calibrated BioBERT
Source: BMC Bioinformatics. 2022 Jan 4;23:4. doi: 10.1186/s12859-021-04504-x (PMC8729035; doi:10.1186/s12859-021-04504-x)
Supplement: Supplementary file 1 — Additional file 1. Appendix data and explanations. [file 12859_2021_4504_MOESM1_ESM.pdf]

Appendix

A.1 BioBERT Training details

We use the following settings to train BioBERT v1.1 based on BERT Base cased using PyTorch 1.4.0. We use gradient accumulation to train with an effective batch size of 64. We train on a Amazon SageMaker P3 instance using a single GPU for approximately 1 hours with early stopping patience.

- Loss function - cross entropy loss
- Optimiser - Adam
- Optimiser Learning rate - .00001,
- Optimiser Weight decay - 0.01,
- Batch size - 8
- Gradient accumulation steps - 8
- Epochs - 1000
- Early stopping patience epochs - 20

The full source code is here `https://github.com/elangovana/large-scale-ptm-ppi`

A.2 BioBERT Token lengths

Here we present the distribution of length of the tokens, as a result of using BERT tokeniser, to tokenise the abstract, see Table A10.

Table A10 The distribution of abstract lengths as a result of using BioBERT tokeniser. Approximately 90% of the unique normalised abstracts is under the max limit of 512.

	Train	Test	Validation
count	1305.00	352.00	200.00
mean	378.21	353.21	375.32
std	95.53	102.59	78.16
min	174.00	175.00	194.00
0%	174.00	175.00	194.00
10%	256.00	209.20	241.90
20%	285.00	250.20	323.00
30%	323.00	296.30	335.00
40%	344.60	323.00	361.80
50%	377.00	348.00	379.00
60%	402.00	376.00	389.00
70%	426.00	414.70	420.90
80%	464.00	447.00	467.00
90%	511.00	496.90	472.00
max	612.00	538.00	496.00

A.3 Performance using lowest loss

The performance on test and validation set where the models in the ensemble are selected based on lowest loss on the validation set. The Table A11 shows the F-score, the reliability diagrams are in Figure A8 and the Figure A9 shows the corresponding confusion matrix.

A.4 Human verified results

Table A11 The performance of ensemble PPI-BioBERT-x10 on the test and validation set where the models are selected based on lowest loss on validation set.

	Test				Validation			
InteractionType	Precision	Recall	F1-score	support	Precision	Recall	F1-score	support
acetylation	0.00	0.00	0.00	1	0.00	0.00	0.00	1
dephosphorylation	0.00	0.00	0.00	6	0.00	0.00	0.00	10
methylation	0.00	0.00	0.00	4	0.00	0.00	0.00	1
phosphorylation	66.67	36.36	47.06	44	56.25	42.86	48.65	21
ubiquitination	0.00	0.00	0.00	1	0.00	0.00	0.00	1
macro avg	13.33	7.27	9.41	56	11.25	8.57	9.73	34
micro avg	66.67	28.57	40.00	56	56.25	26.47	36.00	34

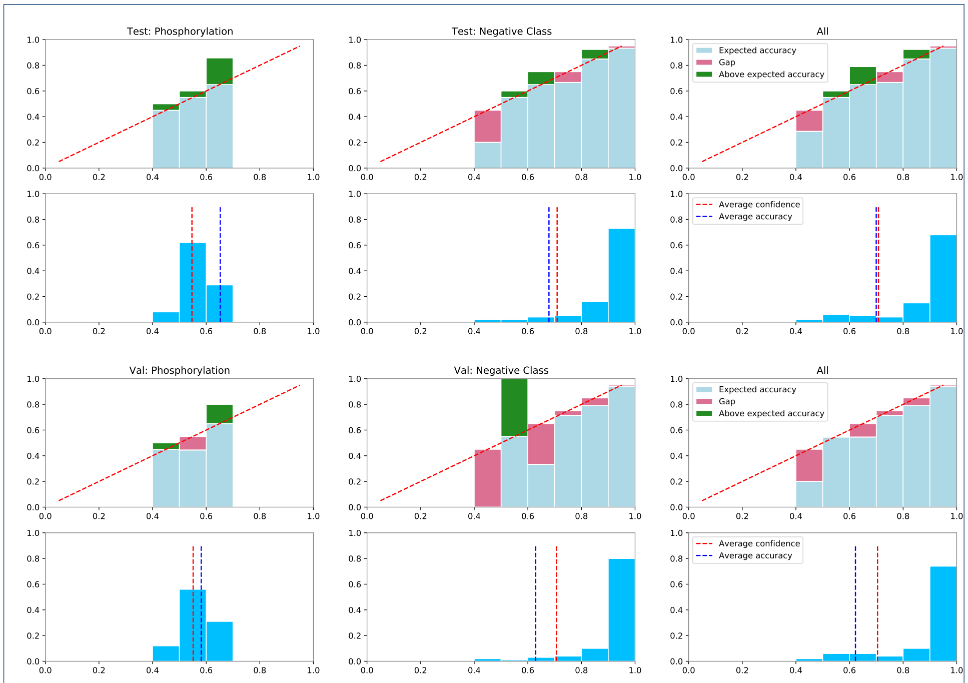

Figure A8 Confidence reliability on the test and validation set using PPI-BioBERT-x10 where the models are selected based on lowest loss on validation set.

Table A12 The distribution of unique normalised protein counts in the abstracts in the test set.

Interaction	count	mean	std	min	25%	50%	75%	max
acetylation	6	3.50	1.38	2.00	3.00	3.00	3.75	6.00
demethylation	2	5.00	0.00	5.00	5.00	5.00	5.00	5.00
dephosphorylation	21	3.62	1.69	2.00	2.00	3.00	5.00	6.00
deubiquitination	1	2.00	nan	2.00	2.00	2.00	2.00	2.00
methylation	11	2.82	0.40	2.00	3.00	3.00	3.00	3.00
phosphorylation	125	3.56	1.95	1.00	2.00	3.00	5.00	9.00
ubiquitination	2	5.00	1.41	4.00	4.50	5.00	5.50	6.00

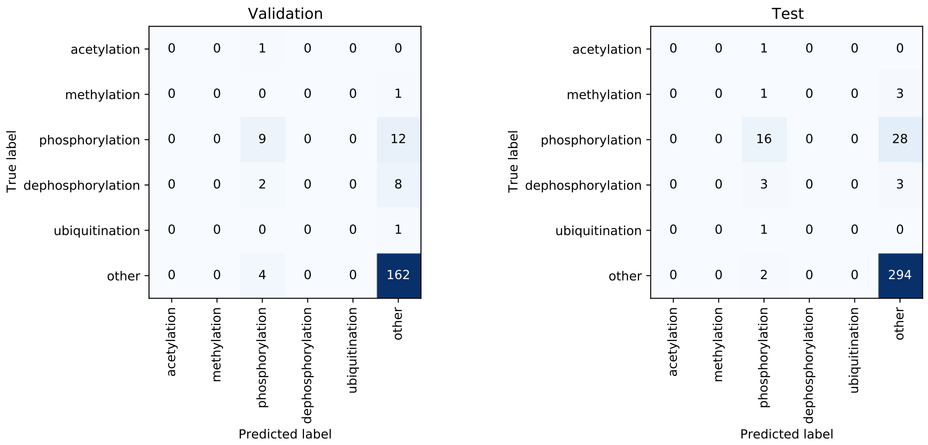

Figure A9 Confusion matrix using PPI-BioBERT-x10 where the models are selected based on lowest loss on validation set.

Table A13 Human verification of randomly sampled subset of 30 PTM-PPI per PTM type. The samples were chosen after confidence calibration and the predictions were present in multiple abstracts within the confidence and standard deviation thresholds.

PubmedId	Participant1 Uniprot	Participant1 Name	Participant2 Uniprot	Participant2 Name	Prediction	Human result
19877273	P23443	S6K1	P42345	mTOR	phosphorylation	Correct
28539327	P01375	TNF	Q13546	RIPK1	phosphorylation	Incorrect - relationship not described
9148953	P06493	Cdc2	P30291	Wee1	phosphorylation	Correct
18028023	P31749	Akt	P42345	mammalian target of rapamycin	phosphorylation	Incorrect - relationship not described
14572154	P31749	PKB/Akt	P60484	PTEN	phosphorylation	Not - sure
1327869	O00757	FBPase-2	Q16875	PFK-2	phosphorylation	Incorrect - relationship not described
22855742	O14757	Checkpoint kinase 1	Q13535	ATR	phosphorylation	Correct
16477614	P23560	Brain-derived neurotrophic factor	P28482	extracellular-signal regulated kinase	phosphorylation	Incorrect - relationship not described
15289331	P31749	Akt	P49841	glycogen synthase kinase-3beta	phosphorylation	Correct
23399841	P01583	IL-1	P10145	CXCL8	phosphorylation	Not - sure
16873552	P12931	Src	P41240	C-terminal Src kinase	phosphorylation	Correct
15994312	Q05397	focal adhesion kinase	Q14289	proline-rich tyrosine kinase-2	phosphorylation	Correct
17190911	P35568	insulin receptor substrate-1	P42336	PI3-K	phosphorylation	Correct
1840422	P17677	B-50	Q92686	neurogranin	phosphorylation	Incorrect - NER
27633668	P20042	eukaryotic translation initiation factor 2	Q9BQI3	heme-regulated inhibitor	phosphorylation	Incorrect - relationship not described
12112022	P31749	PKBalpha	Q14289	Protein kinase B	phosphorylation	Incorrect - relationship not described
23337506	P01138	nerve growth factor	P04629	tropomyosin receptor kinase receptor	phosphorylation	Correct
12759443	O14920	IKK beta	Q04206	p65	phosphorylation	Not - sure
29162743	P16220	CREB	P40763	signal transducer and activator of transcripti...	phosphorylation	Incorrect - NER
9347311	P01019	Angiotensin II	P30556	AT1	phosphorylation	Not - sure
21325496	P24941	cyclin-dependent kinase 2	P38936	p21	phosphorylation	Correct
7763733	P16070	CD44	P60568	IL2	phosphorylation	Incorrect - Not related to PPI
26659448	Q02763	Tek	Q15389	angiopoietin-1	phosphorylation	Correct
20110283	P17676	CCAAT/enhancer binding protein beta	P49841	glycogen synthase kinase 3beta	phosphorylation	Correct
15970650	Q15746	myosin light chain kinase	Q7Z406	myosin	phosphorylation	Correct
29496905	P23443	S6K1)	P35568	insulin receptor substrate-1	phosphorylation	Correct
17402366	P01562	IFN-alpha	P42224	STAT1	phosphorylation	Correct
18198129	P12931	Src	Q05397	FAK	phosphorylation	Correct
17053882	P31749	Akt	P42336	phosphatidylinositol 3-kinase	phosphorylation	Incorrect - relationship not described
24360952	P12931	Src	Q05397	Focal adhesion kinase	phosphorylation	Correct
27411844	P04637	p53	Q9NQR1	SETD8	methylation	Correct
20614940	P04637	p53	Q96KQ7	G9a	methylation	Correct
24151879	P04637	p53	Q96KQ7	G9a	methylation	Correct
25554733	P04637	p53	Q9NQR1	SETD8	methylation	Correct

Table A14 Human verification, without taking into account predictions in multiple abstracts, of randomly sampled subset of 30 PTM-PPI per PTM type. The samples were chosen after confidence calibration.

Pubmed Id	Participant1 Uniprot	Participant1 Name	Participant2 Uniprot	Participant2 Name	Prediction	Human result
22732500	P48431	Sox2	Q01860	Oct3/4	ubiquitination	Incorrect - No trigger word
21492879	P48023	Fas ligand	Q8IXH7	Th1	ubiquitination	Incorrect - No trigger word
19428371	O75943	RAD24	Q99638	RAD9	ubiquitination	Incorrect - No trigger word
17168739	P02786	transferrin receptor	P09544	IRP	ubiquitination	Incorrect - No trigger word
7789533	P19338	C23	Q9Y2Q3	glutathione S-transferase	phosphorylation	Incorrect - No trigger word
19729590	P23443	S6 kinase	P35568	insulin receptor substrate 1	phosphorylation	Incorrect - relationship not described
23478265	O00571	DEAD box protein 3	Q9UHD2	TANK-binding kinase 1	phosphorylation	Incorrect - relationship not described
16365045	O75581	low density lipoprotein receptor-related prote...	P49841	GSK3beta	phosphorylation	Incorrect - relationship not described
19306938	O60500	nephrin	Q9Y5K6	CD2-associated protein	phosphorylation	Incorrect - relationship not described
10720129	P08700	IL3	P36888	FLT3	phosphorylation	Incorrect - relationship not described
27456486	P20701	LFA-1	Q05397	Protein tyrosine kinase 2	phosphorylation	Incorrect - relationship not described
2780290	P19338	nucleolin	P50613	Protein kinase	phosphorylation	Incorrect - NER
17108171	P04629	TrkA	P08138	p75 neurotrophin receptor	phosphorylation	Incorrect - relationship not described
10884023	P16220	Ca2+/cAMP responsive element binding protein	P18509	pituitary adenylate cyclase-activating polypep...	phosphorylation	Incorrect - relationship not described
27118568	O14974	myosin phosphatase-targeting subunit 1	P61586	Rho A	phosphorylation	Incorrect - relationship not described
17353368	Q13094	SH2 domain-containing leukocyte protein of 76 kD	Q92918	hematopoietic progenitor kinase 1	phosphorylation	Correct
20122754	P05067	APP	P45983	JNK	phosphorylation	Correct
23023514	P42574	caspase 3	Q16555	Collapsin response mediator protein-2	phosphorylation	Incorrect - relationship not described
7852364	P78356	PtdIns(4)P 5-kinase	Q9Y2I7	Fab1p	phosphorylation	Incorrect - relationship not described
26464283	P00533	EGFR	P35354	cyclooxygenase-2	phosphorylation	Correct
17211494	Q06124	PTPN11	Q13480	Grb2 associated binder 1	phosphorylation	Incorrect - No trigger word
29514920	P30291	Wee1	Q01850	Cdr2	phosphorylation	Correct
17045592	P49841	GSK-3beta	Q00535	cdk5	phosphorylation	Incorrect - relationship not described
22633971	P35548	Msx2	Q13950	Runx2	phosphorylation	Incorrect - relationship not described
9852063	P42681	tyrosine kinase	P63252	Kir2.1	phosphorylation	Correct
19879273	P31749	Akt	P35222	beta-catenin	phosphorylation	Correct
3014310	P04271	S-100	P50613	protein kinase	phosphorylation	Incorrect - relationship not described
9883577	Q15049	myosin light chain	Q32MK0	MLC kinase	phosphorylation	Incorrect - NER
29162743	P16220	CREB	P40763	signal transducer and activator of transcripti...	phosphorylation	Incorrect - NER
11567986	P62993	Grb2	Q92835	SH2-containing inositol 5'-phosphatase	phosphorylation	Incorrect - relationship not described
30158515	Q00535	CDK5	Q15078	p35	phosphorylation	Incorrect - relationship not described
1707345	O00459	p85 beta	P27986	p85 alpha	phosphorylation	Incorrect - relationship not described
25684187	P01588	Erythropoietin	P17302	connexin43	phosphorylation	Incorrect - relationship not described
25446109	P04792	HSP27	P45983	JNK	phosphorylation	Incorrect - relationship not described
21080372	P08670	eukaryotic translation initiation factor 4A-I ...	Q99873	PRMT1	methylation	Correct
26503212	Q8NCA5	FAM98A	Q99873	PRMT1	methylation	Correct
24412544	Q96L73	NSD1	Q9BQQ3	p65	methylation	Incorrect - relationship not described
25749972	O75164	KDM4A	P42345	mTOR	methylation	Not - sure
25554733	P04637	p53	Q9NQR1	SETD8	methylation	Correct
20615470	Q15047	KMT1E	Q8WTS6	KMT7	methylation	Incorrect - relationship not described
24151879	P04637	p53	Q96KQ7	G9a	methylation	Correct
23469257	O15047	Set1	O75934	Dam1	methylation	Correct
18472002	P04637	p53	P61978	hnRNP K	methylation	Correct
19386523	Q01196	RUNX1	Q06455	ETO	methylation	Incorrect - DNA Methylation
20614940	P04637	p53	Q96KQ7	G9a	methylation	Correct
27411844	P04637	p53	Q9NQR1	SETD8	methylation	Correct
20231378	P11274	B cell antigen receptor	P11912	Igalpha	methylation	Incorrect - NER
24129573	P01562	IFN-	Q01628	Interferon-induced transmembrane protein 3	methylation	Incorrect - relationship not described
26391684	P49757	Numb	Q9NQR1	SET8	methylation	Correct
25350748	Q13283	G3BP1	Q99873	PRMT1	methylation	Correct
26126536	Q99873	PRMT1	Q9HAU4	Smurf2	methylation	Correct
27563394	P35270	SPR	Q8TEK3	disruptor of telomeric silencing 1-like protein	methylation	Incorrect - DNA Methylation
25748791	Q8WWM7	ataxin-2-like	Q99700	ataxin-2	methylation	Incorrect - relationship not described
2967285	Q15746	myosin light chain kinase	Q7Z406	myosin	dephosphorylation	Incorrect - Opposite type
9417127	P01375	tumor necrosis factor-alpha	P29350	SHP-1	dephosphorylation	Incorrect - relationship not described
6245872	P10145	NaF	P50613	protein kinase	dephosphorylation	Incorrect - NER
11264356	Q15181	protein phosphatase 1	Q9BY44	eIF-2alpha	dephosphorylation	Correct
9079814	P35670	WC1	P60568	interleukin (IL)-2	dephosphorylation	Incorrect - relationship not described
8083198	P18031	PTP-1B	P50391	PP-1	dephosphorylation	Incorrect - relationship not described
12853978	P00533	epidermal growth factor receptor	P42574	caspase-3	dephosphorylation	Incorrect - relationship not described
12138178	P42224	Stat1	P42226	Stat6	dephosphorylation	Incorrect - relationship not described
10391142	P01308	insulin	P06213	insulin receptor	dephosphorylation	Incorrect - relationship not described
9119896	P49840	glycogen synthase kinase-3alpha (kinase FA/GSK...	Q9Y2R2	protein tyrosine phosphatase	dephosphorylation	Incorrect - NER
16620785	P31749	Akt	P49840	glycogen synthase kinase-3alpha/beta	dephosphorylation	Correct
7683660	P01308	Insulin	P19338	nucleolin	dephosphorylation	Incorrect - relationship not described
10405762	O14494	PAP2	O43688	hPAP2c	dephosphorylation	Incorrect - relationship not described
10405762	O14494	PAP2	O14495	hPAP2b	dephosphorylation	Incorrect - relationship not described
10496881	P06493	p34cdc2	P30307	cdc25-C	dephosphorylation	Correct
11773439	P17706	TC-PTP	P42229	signal transducer and activator of transcripti...	dephosphorylation	Correct
15823043	P19634	Na(+)/H(+) exchanger isoform 1	Q15181	PP1	dephosphorylation	Correct
15269244	P06213	insulin receptor	P18031	protein-tyrosine phosphatase (PTP) 1B	dephosphorylation	Correct
11495355	P21128	P11	P60903	P10	dephosphorylation	Incorrect - relationship not described
11707519	P16220	cAMP response element-binding protein	P27361	extracellular-signal related kinases 1 and 2	dephosphorylation	Incorrect - No trigger word
1321126	P06213	insulin receptor	P10586	LAR	dephosphorylation	Correct
9989818	P04637	p53	P28749	p107/E2F	dephosphorylation	Incorrect - relationship not described
17046078	P12931	Src	P29350	SHP1	dephosphorylation	Incorrect - relationship not described
9380758	P13569	cystic fibrosis transmembrane conductance regu...	P35813	PP2Calpha	dephosphorylation	Correct
24691491	P20042	eukaryotic translation initiation factor 2	P63000	Rac1	dephosphorylation	Incorrect - relationship not described
11495355	P21128	P11	Q15257	PP2A	dephosphorylation	Incorrect - relationship not described
9989818	P28749	p107/E2F	Q15257	PP2A	dephosphorylation	Correct
11222372	Q15257	PP2A	Q92934	BAD	dephosphorylation	Correct
8550570	Q15181	PP-1	Q15257	phosphatase-2A	dephosphorylation	Correct
14731392	P25440	Brd2	Q92831	PCAF	acetylation	Not - sure
